# Risk Classification Instrument for Orofacial Myofunctional Disorders – ICR/DMO: Reliability (Part III)

**DOI:** 10.1590/1678-7765-2026-0314

**Published:** 2026-08-17

**Authors:** Herick Santos Assis, Íkaro Daniel de Carvalho Barreto, Giédre Berretin-Felix, Andréa Monteiro Correia Medeiros

**Affiliations:** 1 Universidade de São Paulo Faculdade de Odontologia de Bauru Programa de Pós-Graduação em Fonoaudiologia Bauru SP Brasil Universidade de São Paulo, Faculdade de Odontologia de Bauru, Programa de Pós-Graduação em Fonoaudiologia, Bauru, SP, Brasil.; 2 Centro Brasileiro de Pesquisa em Avaliação e Seleção e de Promoção de Eventos Brasília DF Brasil Centro Brasileiro de Pesquisa em Avaliação e Seleção e de Promoção de Eventos (CEBRASPE), Brasília, DF, Brasil.; 3 Universidade de São Paulo Faculdade de Odontologia de Bauru Departamento de Fonoaudiologia Bauru São Paulo Brasil Universidade de São Paulo, Faculdade de Odontologia de Bauru, Departamento de Fonoaudiologia, Bauru, São Paulo, Brasil.; 4 Universidade Federal de Sergipe Departamento de Fonoaudiologia São Cristóvão SE Brasil Universidade Federal de Sergipe, Departamento de Fonoaudiologia, São Cristóvão, SE, Brasil.; 5 Universidade Federal de Sergipe Programa de Pós-Graduação em Ciências Aplicadas à Saúde Lagarto SE Brasil Universidade Federal de Sergipe, Programa de Pós-Graduação em Ciências Aplicadas à Saúde, Lagarto, SE, Brasil.; 6 Universidade Federal de Sergipe Programa de Pós-Graduação em Ciências da Saúde Aracaju SE Brasil Universidade Federal de Sergipe, Programa de Pós-Graduação em Ciências da Saúde, Aracaju, SE, Brasil.; 7 Universidade Federal de Pernambuco Programa de Pós-graduação em Saúde da Comunicação Humana Recife PE Brasil Universidade Federal de Pernambuco, Programa de Pós-graduação em Saúde da Comunicação Humana, Recife, PE, Brasil.

**Keywords:** Mass screening, Risk factors, Reproducibility of results, Infant, Preschool

## Abstract

**Objective:**

To assess the reliability of the Risk Classification Instrument for Orofacial Myofunctional Disorders (ICR/DMO).

**Methodology:**

This reliability study was conducted as part of the broader validation process of the ICR/DMO. Ninety caregivers of infants and preschoolers participated and were allocated into three age groups: G1=30 infants aged 1–5 months; G2=30 infants aged 6–23 months; and G3=30 preschoolers aged 24–71 months. Participants were recruited from two public daycare centers and a university pediatric clinic. The ICR/DMO was administered in person by a single speech-language pathologist (A1) by means of individual interviews with the caregivers, with responses registered in real time and audio-recorded. The audio recordings were analyzed by three additional raters (A2a, A2b, and A2c). After calibration, each rater received 30 audio recordings according to the following group allocation: A2a (G1), A2b (G2), and A2c (G3). Inter-rater agreement (100% of the sample; n=90) and intra-rater reproducibility (20% of the sample; n=18) were assessed using Kendall’s tau (Kendall’s τ). The following interpretation was adopted: weak (<0.4), moderate (0.4–0.7), and strong (>0.7), with p<0.05.

**Results:**

Inter- and intra-rater agreement were observed both for the total scores and for individual instrument items. For the total scores, inter-rater agreement was moderate in G1 (τ=0.576; p=0.001); strong in G2 (τ=0.775; p<0.001), and moderate in G3 (τ=0.583; p<0.001). In all groups, the retest showed strong agreement (G1: τ=0.786; p=0.032; G2: τ=0.881; p=0.002; G3: τ=0.926; p=0.014). For the individual items, both inter-rater and intra-rater agreement showed satisfactory rates of observed agreement.

**Conclusion:**

The ICR/DMO demonstrated evidence of reliability across the three age groups, with inter-rater agreement ranging from moderate to good and intra-rater agreement ranging from good to excellent.

## Graphical abstract

**Figure:**
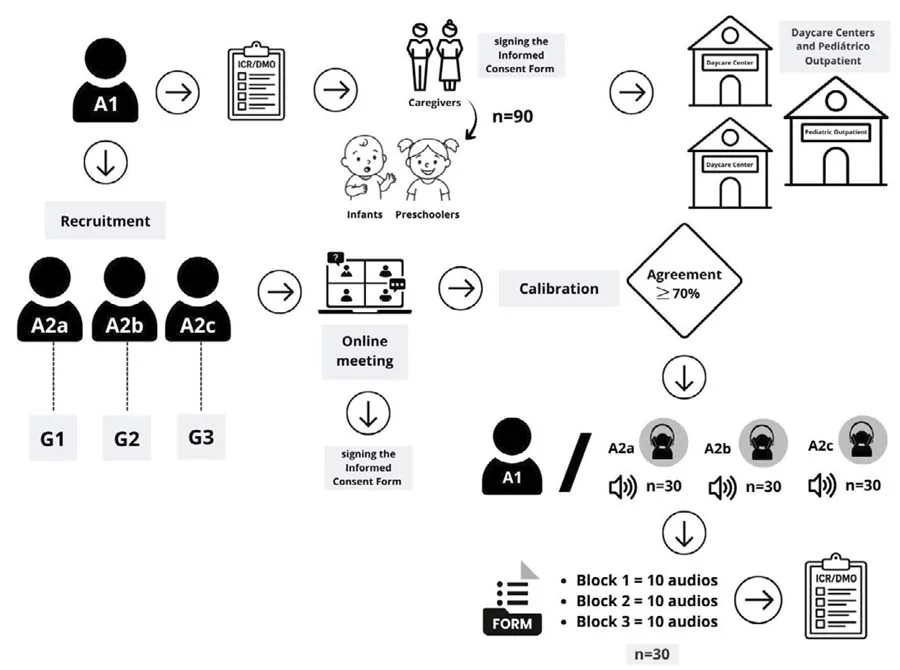

## Introduction

The field of Orofacial Myology (OM) encompasses actions at different levels of healthcare, including the assessment, diagnosis, and rehabilitation of orofacial myofunctional disorders (OMD).^1^ Early identification of risk factors for OMD is essential, since alterations occurring during the first years of life may negatively impact orofacial structures and functions, with consequences for the child’s overall health.^2^

Traditionally, standardized screening instruments have served as strategic tools to support healthcare practices by enabling the early identification of children with signs or a history suggestive of risk, thereby facilitating timely referral for comprehensive clinical evaluation and speech-language pathology intervention.^3,4^ In this context, the Risk Classification Instrument for Orofacial Myofunctional Disorders (ICR/DMO) was developed based on the Clinical History section of the MMBGR Protocol – Infants and Preschoolers.^5^ It is intended as a general screening instrument applicable to both typically developing children and children with atypical developmental conditions.

It is understood that, although risk may already be predicted based on specific clinical factors, the ICR/DMO makes it possible for risk to be quantified via a scoring system that classifies its degree, enabling assessment of not only its presence but also its severity. This feature expands its potential applicability by supporting evidence-based decisions regarding referral for speech-language pathology assessment and intervention.^6^

In pediatric care, which encompasses health promotion and disease prevention as well as the diagnosis and intervention of OM disorders, the importance of using validated protocols recognized as effective tools for the assessment and care of orofacial myofunctional aspects is well established, including those developed for neonatology,^7-9^ infants and preschoolers,^5,10,11^ and children and adults.^12,13^ However, in the context of screening, their application extends beyond the clinical setting. Standardized screening enables the implementation of actions in community-based settings,^3,4,14^ such as outpatient clinics, schools, daycare centers, and other child care environments, thereby broadening the scope and valuing the diverse contributions of speech-language pathology practice.

Therefore, this is a novel instrument intended for use by speech-language pathologists with caregivers of children aged one month to five years and 11 months. It assigns scores that classify the degree of risk for OMD. It should be noted that, in accordance with recommendations and guidelines for test validations in Speech-Language Pathology,^15^ the ICR/DMO has already undergone the stages of validity evidence based on content and response processes. Further validation procedures are therefore warranted.

Accordingly, this study aimed to determine the reliability of the Risk Classification Instrument for Orofacial Myofunctional Disorders (ICR/DMO).

## Methodology

This is descriptive, observational reliability study was conducted with the authorization of the authors of the ICR/DMO and aimed to assess its reliability. The study was conducted within the Communication Processes and Disorders concentration area of the Graduate Program in Speech-Language Pathology at the School of Dentistry of Bauru, University of São Paulo (FOB/USP). The research procedures were developed following approval from the Human Research Ethics Committee (CEP) of the Federal University of Sergipe (UFS), under CAAE: 12529419.6.0000.5546 and opinion number 6.951.517.

The ethical criteria of Resolutions No. 196/96 of the National Ethics Committee in Research (CONEP) and No. 466/12 of the National Health Council (CNS), which govern research involving human beings, were followed. Additionally, the research complied with the Brazilian General Data Protection Law (LGPD), No. 13,709/2018, which regulates the processing of personal data by public or private entities.

The study included speech-language pathologists and caregivers of infants and/or preschoolers who agreed to participate by providing written informed consent. All participants received clear information about the research objectives, potential risks, and benefits. The data were collected and organized according to procedures described in this section.

The inclusion criteria for professionals were being a speech-language pathologist, residing in Brazil, and working in a speech-language pathology setting with infants and preschoolers. For caregivers, the inclusion criteria were answering questions about infants and/or preschoolers without limitations in responding to the questions included in the instrument. The exclusion criteria for professionals were not working with infants and/or preschoolers, residing outside Brazil, having had prior contact with the instrument during previous stages of the validation process, or withdrawing from the study. For caregivers, the exclusion criteria were discomfort or inability to answer the questions included in the instrument.

This study adopted a methodological design based on recognized guidelines for instrument validation,^15,26,28,29^ integrating consolidated psychometric references as well as specific recommendations from Speech-Language Pathology. Based on these references, procedures for reliability estimates were defined, with previously specified criteria and indicators, ensuring rigor, transparency, and reproducibility.

In this step, to determine the case series, the sample size was defined based on statistical precision criteria for Kendall’s tau correlation coefficient (τ), according to the methodology proposed by Bonett and Wright^16^ (2000), and on clinical representativeness criteria (stratification). The precision of τ was defined by the maximum acceptable width for the 95% confidence Interval (ω), considering the need to ensure a sample size that met the stratification requirements. An expected Kendall’s tau (𝜏) of 0.70 was adopted, representing a strong association and considered clinically acceptable for reliability.^17,18^ To ensure a minimum sample size (n) that enabled representative and robust subgroup analysis, a precision criterion was adopted in which the width of the confidence interval (CI) should not exceed ω=0.32. The minimum n required to achieve this precision was calculated using the following equation, which is a rearrangement of the asymptotic standard error formula proposed by Bonett and Wright^16^ (2000) and cited in a recent study.^19^


$$n\approx \frac{{\left(2\cdot Z_{1-\frac{\alpha }{2}}\cdot c\cdot \left(1-\tau_{0}^{2}\right)\right)}^{2}}{\omega }+b$$

In which: $Z_{1-\frac{\alpha }{2}}=1.96$ (95% confidence level); 𝜏_0_=0.70 (expected Kendall’s tau); and ω=0.275 (maximum adopted CI width). The constants c≈0.661 and b=4 are adjustment factors introduced to adapt the parametric correlation formula (Pearson’s correlation) to the non-parametric Kendall’s tau coefficient, correcting for variance and small-sample bias.

Applying the parameters, $n\approx \frac{{\left(2\cdot 1.96\cdot 0.661\cdot \left(1-0.7^{2}\right)\right)}^{2}}{0.275}+4\approx 28.80$ participants. The statistical calculation indicates that n≈28.80 participants is the minimum necessary to guarantee the adopted precision criterion (ω≤0.275). According to the rounding rule for sampling plans (ceiling function), this value was rounded up, resulting in a minimum of n=29 participants per stratum. To ensure a complete and balanced sample, as well as the clinical representativeness of each stratum (younger infants, older infants, and preschoolers), a final sample of n=30 participants per group was adopted. This sample size met the statistical requirement (n≥28.80) and was therefore used in each stratum, ensuring an independent reliability analysis for each age group, with a total of n=90 participants.

Thus, 90 caregivers of infants and/or preschoolers participated in this study and were distributed into three age groups: 30 infants aged 1–5 months (G1), 30 infants aged 6–23 months (G2), and 30 preschoolers aged 24–71 months (G3). Participants were recruited from two public daycare centers in the municipality of Barra dos Coqueiros, Sergipe State, and from the pediatric outpatient clinic of the University Hospital of UFS.

It is important to note that all caregivers signed the informed consent form and the authorization form for the use of their image and testimony. The inclusion and exclusion criteria were applied to both the caregivers of the infants and/or preschoolers and the three recruited raters.

The ICR/DMO was administered in person by a single speech-language pathologist (A1) by means of individual interviews with caregivers, with responses registered in real time and the interviews audio-recorded simultaneously. The interviews were recorded without editing and made available for analysis by three experienced and calibrated raters (speech-language pathologists), identified as A2a, A2b, and A2c. The recordings were stored in a cloud drive according to the ICR/DMO application, which consists of 20 items classified as Low Risk (0), Risk (1), and High Risk (2).

It is noteworthy that the three raters (A2a, A2b, A2c) each have five years of experience in OM, including professional experience working with infants and preschoolers. Specifically, A2a holds a postgraduate degree in Neonatal Speech-Language Pathology and is a co-author of an assessment protocol for infants; A2b is also a co-author of a protocol for this age group and holds a master’s degree in OM; and A2c holds a master’s degree in Om and coordinates speech-language pathology services for children (**Figure 1**). Furthermore, all three have publications related to pediatric practice in MO. This composition ensures clinical and academic expertise aligned with the scope of the instrument.

**Figure 1:**
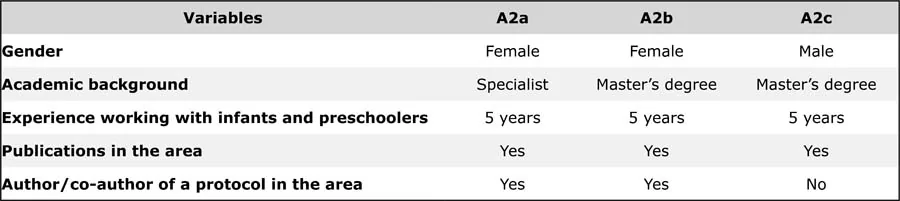
Characterization of the panel of evaluators in the reliability stage of the Risk Classification Instrument for Orofacial Myofunctional Disorders – ICR/DMO.

A2a = Rater 2a; A2b = Rater 2b; A2c = Rater A2c.

### Application of the ICR/DMO by rater 1 (A1) – test and retest

The ICR/DMO was applied *in situ* (at the daycare centers and outpatient clinic) by a single speech-language pathologist (A1). Support was provided by the research team (members of the Orofacial Motricity Study and Research Group – GEPMO/UFS), which includes undergraduate, master’s, and doctoral students, as the research is part of an umbrella project including several research areas under the coordination of the supervisor of this study.

The research team was calibrated with prior training provided by A1, with instructions on how to assist the researcher. Before data collection began, the team members were given the complete version of the ICR/DMO for reference. Furthermore, they received guidance on how to introduce themselves to the participants and how to assist the researcher during the administration of the instrument.

The ICR/DMO was administered by A1 to the caregivers by means of individual interviews, with responses recorded in real time and audio recorded using a mobile phone (Apple iPhone™), creating a raw audio database. To standardize the procedures and maintain methodological rigor, all surveys/recordings took place in the same room at each daycare center and in the same room at the outpatient clinic. During data collection, A1 and the caregiver remained seated, using individual wireless lapel microphones (K9 type), with all necessary materials available on the table.

For the retest, 20% of the total sample (n=18) was randomly selected, corresponding to six cases per age group (G1, G2, and G3). Intra-rater reassessment was conducted by A1 30 days after the initial assessment, based on the audio recordings obtained during the first application with the caregivers. The use of audio recordings ensured exposure to the same reports, preserving the comparability of the screening. The 30-day interval was adopted to minimize memory effects, as recommended for reliability studies.^8^ It is noteworthy that the scores were assigned according to the same criteria and instructions of the ICR/DMO, aiming to estimate the temporal stability (intra-rater) of the response pattern.

Assessment by raters A2a, A2b, and A2c (regarding the ICR/DMO assessments based on listening to the recorded audio).

Each rater underwent a calibration process. The first step of this process took place virtually, during an online meeting, in which the ICR/DMO was presented without reporting or discussing A1’s experiences with the administration of the instrument to avoid response bias. After all questions had been addressed, the raters agreed to participate by signing the Informed Consent Form.

During the second step of calibration, each pair of raters (A1/A2a, A1/A2b, and A1/A2c) applied the ICR/DMO to the same subject (by listening to the recorded audios), without communicating with each other, and a minimum agreement of 70% had to be achieved in five consecutive cases. The study cases were released for analysis only after the raters reached this level of agreement. Subsequently, the raters received 30 audio recordings according to the age-group allocation: A2a (G1), A2b (G2), and A2c (G3).

It should be noted that, since A1 had already applied the instrument *in situ* and recorded responses on paper forms, the same data had to be transferred to electronic forms, which were also used by the other raters to record their responses item by item for each infant or preschooler while listening to the corresponding audio recordings, ensuring accuracy. The electronic forms were developed by A1 and organized into blocks corresponding to the age groups.

For each age group (G1, G2, and G3), three electronic forms were made available, each containing a block of 10 cases, totaling 30 cases. Each form contained an image of the ICR/DMO and presented its items as response options. The questions corresponded to the original version instrument and were completed according to the information provided in the recorded audios of each of the 10 cases in the respective block.

Raters A2a, A2b, and A2c accessed the audio files stored in a cloud repository, identified by unique codes and organized according to the same blocks to standardize the workflow. Thus, for each block, the raters listened to the corresponding audio files and recorded their responses on the homologous form in real time, ensuring correct case-to-form matching, reducing errors, and preserving traceability.

### Statistical analysis – reliability

Throughout this manuscript, the following terminology is adopted, consistent with COSMIN recommendations:^28^ reliability refers to the overall property of an instrument to produce consistent measurements. It encompasses both inter-rater reliability (agreement between different raters scoring the same cases) and test-retest reliability (also referred to as intra-rater reproducibility, i.e., the temporal stability of scores assigned by the same rater). The term agreement is used for absolute concordance between scores. The term reproducibility is used specifically as a synonym for test-retest reliability.

All records were analyzed individually and confidentially, paired with the main rater (A1). Inter-rater agreement (100% of the sample; n=90) and intra-rater reproducibility (20% of the sample; n=18; six cases per group) were assessed using Kendall’s τ, with the following interpretation of values: weak (<0.4), moderate (0.4–0.7), and strong (>0.7).^20,21^

### Agreement Assessment Method

Inter-rater agreement and intra-rater reproducibility were assessed using Kendall’s τ. This coefficient was chosen because it is appropriate for ordinal variables, as is the case with the outcomes used in this study, which assume values on discrete scales (e.g., 0 = absence, 1 = mild, 2 = severe).

Unlike variance-based coefficients, such as the intraclass correlation coefficient (ICC), Kendall’s τ assesses the agreement between rankings of two sets of measurements based on the proportion of concordant and discordant pairs. It is robust to data distribution and suitable for asymmetrical scales with few levels, avoiding unnecessary parametric assumptions.^22^

To complement the primary analysis and address known limitations of single-coefficient reliability assessment in ordinal scales, the following sensitivity analyses were performed: (i) Quadratic-Weighted Kappa (κw),^22^ which assigns greater penalty to disagreements according to category distance; (ii) Gwet’s AC2 coefficient with quadratic weights,^23,37^ specifically chosen for its robustness to the prevalence paradox phenomenon, which may lead conventional chance-corrected coefficients to underestimate agreement in items where one category dominates the marginal distribution;^23^ and (iii) for the sum of scores, the ICC using the two-way random-effects, single-rater, absolute-agreement formulation—ICC(2,1)—was included,^17^ since the summed score is quasi-continuous and meets the assumptions required for ICC estimation.

### Justification for not using ICC or Kappa

The use of the ICC was discarded in this analysis, despite being widely used for reliability assessment, because its formulation assumes continuous data and approximately normal distributions. In the analyzed data, the outcomes were ordinal with low variability and frequently showed values concentrated at a single point on the scale, which violates ICC assumptions and may make the coefficient unstable or undefined in several cases.

Alternatives such as Cohen’s Kappa (and its weighted version) were also considered inadequate due to the presence of categories with unbalanced marginal frequencies and Kappa’s sensitivity to prevalence bias. In scenarios with high agreement and low variability, Kappa tends to underestimate the observed agreement, making its accurate interpretation difficult.^23^

Cohen’s Kappa and its weighted version were not adopted as primary measures due to the documented sensitivity of these coefficients to prevalence bias and unbalanced marginal frequencies, conditions observed in several ICR/DMO items. In such scenarios, Kappa is known to underestimate observed agreement—the so-called Kappa paradox.^37^ Nonetheless, given that this issue was raised during peer review, κw was calculated and is reported in Tables 1–6 as a sensitivity analysis, alongside Gwet’s AC2 coefficient, which was specifically developed to address this paradox.^23,37^

### Limitations of Kendall’s τ and statistical handling

Kendall’s τ cannot be calculated when there is no variability in at least one of the compared measures (i.e., when one of the raters assigns exactly the same value to all cases). In such cases, statistical agreement is indeterminate because it is not possible to establish ordering relationships between pairs. However, recognizing that identical values between raters reflect, from a descriptive perspective, complete uniformity between judgments, the practice of recording such situations as “complete response uniformity (identical values, no variance to estimate τ)” was adopted. The analysis was complemented by the percentage of gross agreement, defined as the proportion of observations in which raters assigned exactly the same value.

This indicator, although lacking inferential interpretation, offers an intuitive measure of similarity between evaluations and was reported in cases where Kendall’s τ could not be calculated. In this study, all statistical analyses were performed using the R software (version 4.3.2)^24^ and the adopted significance level was 5%.

For ICC(2,1), interpretation followed Koo and Li^17^ (2016): poor (<0.50), moderate (0.50–0.75), good (0.75–0.90), and excellent (>0.90). Additionally, to address the methodological distinction between association and absolute agreement, Bowker’s test of symmetry was applied to each item in the inter-rater analysis. This test evaluates whether the off-diagonal frequencies of the rater-by-rater contingency table are symmetric; a non-significant result (p > 0.05) indicates the absence of systematic directional bias between raters, supporting the interpretation that the agreement measured by Kendall’s τ reflects concordance rather than mere association.

### Application time of the ICR/DMO

The application/recording times were initially converted to seconds and then subjected to descriptive analysis using Jamovi statistical software (version 2.3).^25^ Measures of central tendency (mean) and dispersion (standard deviation) were calculated to characterize the average time and variability of recordings in each group. For presentation purposes, the values were reconverted and expressed in minutes and seconds.

## Results

The results of this study included the reliability analysis of the ICR/DMO. This analysis enabled the verification of the stability and consistency of responses, both intra- and inter-rater, ensuring the reproducibility of the instrument under similar conditions. Thus, the combination of these steps represents an advance in the psychometric evidence of the ICR/DMO, reinforcing its scientific applicability and in different speech-language pathology contexts.

The reliability of the ICR/DMO applications was examined from inter- and intra-rater perspectives, both in grouped form (sums of assigned scores) and stratified form (each item of the instrument). Kendall’s τ coefficient and gross agreement (%) were used as metrics, as shown in the corresponding tables.

### Grouped form (sums of assigned scores)

Regarding the sum of scores, moderate to strong correlations were found in the inter-rater and retest (intra-rater) assessments. For infants (G1), inter-rater agreement was moderate (τ=0.576; p=0.0001) and strong in the retest (τ=0.786; p=0.032). Among infants (G2), agreement was strong in both analyses (inter-rater: τ=0.775; p<0.0001; retest: τ=0.881; p=0.002). In preschoolers (G3), inter-rater agreement was moderate (τ=0.583; p<0.0001), whereas retest agreement was strong (τ=0.926; p=0.014). These results demonstrate the stability and consistency of the ICR/DMO across different age groups.

The ICC(2,1) with absolute agreement reinforced the Kendall results: inter-rater ICC was moderate in G1 (0.633; 95% CI: 0.362–0.806) and G3 (0.685; 95% CI: 0.435–0.837), and good in G2 (0.896; 95% CI: 0.795–0.949). Intra-rater ICC was good in G1 (0.835; 95% CI: 0.300–0.975) and excellent in G2 (0.983; 95% CI: 0.900–0.997) and G3 (0.914; 95% CI: 0.391–0.988). The wider confidence intervals observed in the G1 and G3 retests reflect the limited intra-rater sample size (n=6 per group, 20% subsampling).

### Stratified form (each item of the instrument)

In Tables 1–6, κw and Gwet’s AC2 are presented alongside Kendall’s τ and gross agreement (%C). For items in which τ could not be calculated due to zero variance or κw was affected by the prevalence paradox, AC2 consistently yielded values above 0.80, supporting the interpretation of substantial item-level agreement even under degenerate marginal distributions.

Bowker’s test of symmetry indicated absence of systematic bias between raters in the vast majority of items: only five of 43 testable items showed significant asymmetry (p<0.05)—none in G1 (0 of seven), one in G2 (one of 18: “Gestational and perinatal complications”), and four in G3 (four of 18: “Complaint,” “Gestational and perinatal complications,” “Health treatments,” “Oral habits”). This pattern supports the interpretation that the agreement captured by Kendall’s τ reflects concordance rather than mere association, with localized exceptions concentrated in items where caregivers’ reports vary widely in detail and granularity.

In G1 (infants aged 1–5 months), a generally favorable pattern of inter-rater agreement was observed. Among the 10 items, five were significant: “Complaint,” “Gestational and perinatal complications,” “Health problems,” “Sleep,” and “Feeding development – Breastfeeding.” Three items showed complete response uniformity (100% gross agreement, no variance to estimate τ): “Motor development,” “Motor difficulties,” and “Respiratory problems.” The item “Health treatments” did not reach significance but obtained 73.3% gross agreement. In “Family history (regarding the complaint),” no variance was observed despite the high gross agreement (96.7%) (**Table 1)**.

**Table 1:** Inter-rater agreement (infants aged 1-5 months).

Variable	τ	p	%C	κw	p	AC2	p
1. Complaint	1.00	**0.025**	0.833	**0.769**	**0.014**	**0.941**	**<0.001**
2. Family history (regarding the complaint)	*		1.000	—	—	**1.000**	**<0.001**
3. Gestational and perinatal complications	1.00	**0.025**	1.000	**1.000**	**0.014**	**1.000**	**<0.001**
4. Motor development*	*		1.000	—	—	**1.000**	**<0.001**
5. Motor difficulties	*		1.000	—	—	**1.000**	**<0.001**
6. Health problems	0.87	**0.031**	0.833	**0.857**	**0.029**	**0.880**	**0.001**
7. Respiratory problems	**		0.833	0.000	1.000	**0.951**	**<0.001**
8. Sleep	1.00	**0.025**	1.000	**1.000**	**0.014**	**1.000**	**<0.001**
9. Health treatments	1.00	**0.025**	1.000	**1.000**	**0.014**	**1.000**	**<0.001**
10. Feeding development – Breastfeeding	1.00	**0.014**	1.000	**1.000**	**0.014**	**1.000**	**<0.001**

N – Valid data. τ – Kendall’s correlation. %C – Gross agreement. * Complete response uniformity (identical values, no variance to estimate τ). ** No variance in one of the raters – statistical agreement cannot be calculated.

For G1, the results indicate intra-rater stability across all items. Reproducibility with 100% gross agreement was observed for seven items: “Family history,” “Gestational and perinatal complications,” “Motor development,” “Motor difficulties,” “Sleep,” “Health treatments and development of feeding – Breastfeeding.” Some items showed high agreement (83.3%): “Complaint,” “Health problems,” and “Respiratory problems,” the latter also showing an absence of variance (**Table 2**).

**Table 2:** Intra-rater reproducibility (infants aged 1-5 months).

Variable	τ	p	%C	κw	p	AC2	p
1. Complaint	0.411	**0.023**	0.833	0.351	0.054	**0.913**	**<0.001**
2. Family history (regarding the complaint)	*		0.967	0.000	1.000	**0.991**	**<0.001**
3. Gestational and perinatal complications	0.771	**<0.001**	0.833	**0.798**	**<0.001**	**0.903**	**<0.001**
4. Motor development*	**		1.000	—	—	**1.000**	**<0.001**
5. Motor difficulties	**		1.000	—	—	**1.000**	**<0.001**
6. Health problems	0.424	**0.016**	0.633	**0.586**	**0.001**	**0.835**	**<0.001**
7. Respiratory problems	**		1.000	—	—	**1.000**	**<0.001**
8. Sleep	0.462	**0.012**	0.833	**0.495**	**0.007**	**0.796**	**<0.001**
9. Health treatments	0.243	0.184	0.733	0.225	0.202	**0.867**	**<0.001**
10. Feeding development – Breastfeeding	0.914	**<0.001**	0.933	**0.932**	**<0.001**	**0.956**	**<0.001**

N – Valid data. τ – Kendall’s correlation. %C – Gross agreement. * No variance in one of the raters – statistical agreement cannot be calculated. ** Complete response uniformity (identical values, no variance to estimate τ).

In G2 (infants aged 6–23 months), a generally favorable pattern of inter-rater agreement was observed. Among the 20 items, statistical significance was observed for the majority, and gross agreement was high, with minimum of 66.7% for “Gestational and perinatal complications.” It should be noted that items without statistical significance still showed high agreement, such as “Feeding” (90%), “Speech” (93.3%), and “Voice” (93.3%) (**Table 3**).

**Table 3:** Inter-rater agreement (Infants aged 6-23 months).

Variable	τ	p	%C	κw	p	AC2	p
1. Complaint	0.525	**0.004**	0.867	0.324	0.072	**0.875**	**<0.001**
2. Family history (regarding the complaint)	0.393	**0.031**	0.900	**0.706**	**<0.001**	**0.970**	**<0.001**
3. Gestational and perinatal complications	0.504	**0.003**	0.667	**0.436**	**0.003**	**0.639**	**<0.001**
4. Motor development*	0.698	**<0.001**	0.933	**0.667**	**<0.001**	**0.945**	**<0.001**
5. Motor difficulties	0.813	**<0.001**	0.933	**0.706**	**<0.001**	**0.947**	**<0.001**
6. Health problems	0.738	**<0.001**	0.867	**0.756**	**<0.001**	**0.864**	**<0.001**
7. Respiratory problems	0.972	**<0.001**	0.967	**0.972**	**<0.001**	**0.977**	**<0.001**
8. Sleep	0.820	**<0.001**	0.933	**0.814**	**<0.001**	**0.888**	**<0.001**
9. Health treatments	0.713	**<0.001**	0.767	**0.662**	**<0.001**	**0.884**	**<0.001**
10. Feeding development – Breastfeeding	0.922	**<0.001**	0.933	**0.920**	**<0.001**	**0.962**	**<0.001**
11. Feeding – Introduction of utensils, flavors, and textures	0.810	**<0.001**	0.900	**0.757**	**<0.001**	**0.963**	**<0.001**
12. Current diet – type and acceptance	1.000	**<0.001**	0.967	**0.795**	**<0.001**	**0.991**	**<0.001**
13. Current Feeding – Type and Acceptance (Consistency, Texture and Food Groups)	0.630	**0.001**	0.933	**0.630**	**0.001**	**0.980**	**<0.001**
14. Eating meals	*		0.900	0.000	—	**0.917**	**<0.001**
15. Chewing	1.000	**<0.001**	1.000	**1.000**	**<0.001**	**1.000**	**<0.001**
16. Swallowing	0.464	**0.012**	0.933	**0.464**	**0.011**	**0.981**	**<0.001**
17. Oral habits	0.894	**<0.001**	0.967	**0.889**	**<0.001**	**0.988**	**<0.001**
18. Speech	*		0.933	0.000	—	**0.982**	**<0.001**
19. Hearing	1.000	**<0.001**	1.000	**1.000**	**<0.001**	**1.000**	**<0.001**
20. Voice	*		0.933	0.000	1.000	**0.955**	**<0.001**

N – Valid data. τ – Kendall’s correlation. %C – Gross agreement. * No variance in one of the raters – statistical agreement cannot be calculated. ** Complete response uniformity (identical values, no variance to estimate τ).

For G2, the results indicate intra-rater stability. Most items showed significance and 100% agreement, with complete response uniformity observed for 11 of the 20 items on the instrument. It should be noted that the item “Gestational and perinatal complications” did not show a statistical significance but registered high gross agreement (83.3%) (**Table 4**).

**Table 4:** Intra-rater reproducibility (infants aged 6-23 months).

Variable	τ	p	%C	κw	p	AC2	p
1. Complaint	*		1.000	—	—	**1.000**	**<0.001**
2. Family history (regarding the complaint)	*		1.000	—	—	**1.000**	**<0.001**
3. Gestational and perinatal complications	0.707	0.114	0.833	0.667	0.083	**0.919**	**<0.001**
4. Motor development*	1.000	**0.025**	1.000	**1.000**	**0.014**	**1.000**	**<0.001**
5. Motor difficulties	*		1.000	—	—	**1.000**	**<0.001**
6. Health problems	1.000	**0.025**	1.000	**1.000**	**0.014**	**1.000**	**<0.001**
7. Respiratory problems	1.000	**0.025**	1.000	**1.000**	**0.014**	**1.000**	**<0.001**
8. Sleep	1.000	**0.025**	1.000	**1.000**	**0.014**	**1.000**	**<0.001**
9. Health treatments	0.905	**0.034**	0.833	**0.800**	**0.028**	**0.903**	**0.001**
10. Feeding development – Breastfeeding	1.000	**0.012**	1.000	**1.000**	**0.014**	**1.000**	**<0.001**
11. Feeding – Introduction of utensils, flavors, and textures	*		1.000	—	—	**1.000**	**<0.001**
12. Current diet – type and acceptance	*		1.000	—	—	**1.000**	**<0.001**
13. Current Feeding – Type and Acceptance (Consistency, Texture and Food Groups)	1.000	**0.025**	1.000	**1.000**	**0.014**	**1.000**	**<0.001**
14. Eating meals	*		1.000	—	—	**1.000**	**<0.001**
15. Chewing	*		1.000	—	—	**1.000**	**<0.001**
16. Swallowing	*		1.000	—	—	**1.000**	**<0.001**
17. Oral habits	1.000	**0.025**	1.000	**1.000**	**0.014**	**1.000**	**<0.001**
18. Speech	*		1.000	—	—	**1.000**	**<0.001**
19. Hearing	*		1.000	—	—	**1.000**	**<0.001**
20. Voice	*		1.000	—	—	**1.000**	**<0.001**

N – Valid data. τ – Kendall’s correlation. %C – Gross agreement. * Complete response uniformity (identical values, no variance to estimate τ).

In G3 (preschoolers aged 24–71 months), a generally favorable pattern of inter-rater agreement was observed. Most items showed statistically significant values, with complete response uniformity for “Motor Difficulty.” The items “Current Feeding – Type and Acceptance (Consistency, Texture, and Food Groups)” and “Hearing” did not show statistically significant values, but their gross agreements remained high (83.3%) (**Table 5**).

**Table 5:** Inter-rater agreement (preschoolers aged 24-71 months).

Variable	τ	p	%C	κw	p	AC2	p
1. Complaint	0.588	**0.001**	0.667	**0.530**	**0.002**	**0.821**	**<0.001**
2. Family history (regarding the complaint)	0.608	**0.001**	0.900	**0.617**	**<0.001**	**0.932**	**<0.001**
3. Gestational and perinatal complications	0.592	**0.001**	0.833	**0.658**	**<0.001**	**0.940**	**<0.001**
4. Motor development*	0.874	**<0.001**	0.900	**0.772**	**<0.001**	**0.966**	**<0.001**
5. Motor difficulties	*		1.000	—	—	**1.000**	**<0.001**
6. Health problems	0.419	**0.022**	0.800	**0.434**	**0.005**	**0.932**	**<0.001**
7. Respiratory problems	0.852	**<0.001**	0.933	**0.903**	**<0.001**	**0.973**	**<0.001**
8. Sleep	0.685	**<0.001**	0.800	**0.634**	**<0.001**	**0.890**	**<0.001**
9. Health treatments	0.417	**0.023**	0.800	**0.536**	**0.001**	**0.934**	**<0.001**
10. Feeding development – Breastfeeding	0.496	**0.005**	0.700	**0.490**	**0.005**	**0.790**	**<0.001**
11. Feeding – Introduction of utensils, flavors, and textures	0.183	0.319	0.833	0.111	0.502	**0.916**	**<0.001**
12. Current diet – type and acceptance	0.736	**<0.001**	0.867	**0.676**	**<0.001**	**0.956**	**<0.001**
13. Current Feeding – Type and Acceptance (Consistency, Texture and Food Groups)	0.638	**<0.001**	0.900	**0.769**	**<0.001**	**0.966**	**<0.001**
14. Eating meals	0.638	**<0.001**	0.900	**0.769**	**<0.001**	**0.966**	**<0.001**
15. Chewing	1.000	**<0.001**	1.000	**1.000**	**<0.001**	**1.000**	**<0.001**
16. Swallowing	0.802	**<0.001**	0.967	**0.783**	**<0.001**	**0.990**	**<0.001**
17. Oral habits	0.407	**0.022**	0.500	**0.331**	**0.028**	**0.548**	**0.001**
18. Speech	0.517	**0.004**	0.733	**0.580**	**0.001**	**0.859**	**<0.001**
19. Hearing	0.224	0.223	0.833	0.130	0.356	**0.916**	**<0.001**
20. Voice	0.367	**0.046**	0.867	**0.323**	**0.019**	**0.931**	**<0.001**

N – Valid data. τ – Kendall’s correlation. %C – Gross agreement. * Complete response uniformity (identical values, no variance to estimate τ).

For G3, the results indicate high intra-rater stability. Most items showed significance and 100% agreement, with complete response uniformity for seven of the 20 items on the instrument. The item “Eating meals” did not show statistical significance, but registered high gross agreement (83.3%) (**Table 6**).

**Table 6:** Intra-rater reproducibility (preschoolers aged 24-71 months).

Variable	τ	p	%C	κw	p	AC2	p
1. Complaint	0.539	0.206	0.667	0.455	0.184	**0.838**	**0.001**
2. Family history (regarding the complaint)	1.000	**0.025**	1.000	**1.000**	**0.014**	**1.000**	**<0.001**
3. Gestational and perinatal complications	1.000	**0.025**	1.000	**1.000**	**0.014**	**1.000**	**<0.001**
4. Motor development*	1.000	**0.025**	1.000	**1.000**	**0.014**	**1.000**	**<0.001**
5. Motor difficulties	*		1.000	—	—	**1.000**	**<0.001**
6. Health problems	1.000	**0.025**	1.000	**1.000**	**0.014**	**1.000**	**<0.001**
7. Respiratory problems	1.000	**0.025**	1.000	**1.000**	**0.014**	**1.000**	**<0.001**
8. Sleep	**		0.833	0.000	1.000	**0.803**	**0.017**
9. Health treatments	0.632	0.157	0.833	0.571	0.121	**0.933**	**<0.001**
10. Feeding development – Breastfeeding	1.000	**0.014**	1.000	**1.000**	**0.014**	**1.000**	**<0.001**
11. Feeding – Introduction of utensils, flavors, and textures	1.000	**0.025**	1.000	**1.000**	**0.014**	**1.000**	**<0.001**
12. Current diet – type and acceptance	*		1.000	—	—	**1.000**	**<0.001**
13. Current Feeding – Type and Acceptance (Consistency, Texture and Food Groups)	*		1.000	—	—	**1.000**	**<0.001**
14. Eating meals	**		0.833	0.000	1.000	**0.951**	**<0.001**
15. Chewing	*		1.000	—	—	**1.000**	**<0.001**
16. Swallowing	*		1.000	—	—	**1.000**	**<0.001**
17. Oral habits	0.632	0.157	0.833	0.571	0.121	**0.933**	**<0.001**
18. Speech	1.000	**0.025**	1.000	**1.000**	**0.014**	**1.000**	**<0.001**
19. Hearing	*		1.000	—	—	**1.000**	**<0.001**
20. Voice	*		1.000	—	—	**1.000**	**<0.001**

N – Valid data. τ – Kendall’s correlation. %C – Gross agreement. * Complete response uniformity (identical values, no variance to estimate τ). ** No variance in one of the raters – statistical agreement cannot be calculated.

Finally, the application/recording time of the 30 cases from each age group, performed by A1, was recorded (Appendix 1, https://data.scielo.org/file.xhtml?persistentId=doi:10.48331/SCIELODATA.0PPVRC/1ZOAVU). The analysis by group showed that G1 had a shorter average time (1 min 26 s) and less variability (SD=30 s; range: 0 min 51 s–2 min 47 s), indicating greater homogeneity within the group. G2 had a longer average time (4 min 06 s) and intermediate variability (SD=1 min 18 s; range: 2 min 03 s–7 min 47 s). G3 had an average of 3 min 50 s, with greater dispersion (SD=1 min 26 s; range 1 min 47 s–6 min 39 s), showing greater individual heterogeneity within the group. The findings indicate that the application time is brief across all age groups, with greater consistency among younger infants and increased variability with age.

Overall, the reliability analyses support the use of the ICR/DMO, with the caveat that some items showed limited variability, which constrained statistical estimation and required interpretation using complementary coefficients. Regarding the application time measured from the 90 audios recorded by A1, there was agreement with the result obtained in the validation step of the response processes, in which 80% of speech-language pathologists applied the ICR/DMO in less than 20 minutes.

## Discussion

This study presents the reliability^7,8,11^ evidence of the ICR/DMO, addressing the need to advance the validation steps of an instrument^15^ by means of consolidated and reliable methodological approaches.^26^

Reliability constitutes a fundamental domain in psychometric evaluation, as it expresses the degree to which the items of an instrument consistently measure the proposed attribute while minimizing the influence of random errors.^27^ In this study, this aspect was examined with test–retest reproducibility^28^ to estimate whether the factors assessed by the ICR/DMO remained stable when reapplied under similar conditions. The interval between applications represents a critical variable in this type of analysis: very long intervals may introduce changes related to child development, whereas intervals that are too short may overestimate agreement due to the memory effect. The choice of a 30-day interval^8^ was a positive aspect, as it balanced these possibilities and was also compatible with the data collection dynamics in daycare centers and outpatient clinics, conducted by the main researcher, ensuring greater reliability of the instrument’s temporal stability estimates.^29^

### Grouped form (sums of assigned scores)

The reliability results obtained by summing the scores showed moderate to strong correlations in both inter-rater analyses and intra-rater retests across all age groups. This pattern indicates that the ICR/DMO presents adequate stability and consistency^20,21^ for application in different real-world settings by speech-language pathologists.

The data obtained in G1 reflect greater clinical variability in this age group, whereas G2 suggests greater robustness of the instrument when applied to children with more advanced development. In G3, the results indicated that, despite the functional complexity of this age group, the ICR/DMO maintained satisfactory rates.^30^

The findings from this stage corroborate methodological recommendations for validating instruments in health, in which reliability is considered an essential criterion to ensure reproducibility and measurement precision.^31^ In speech-language pathology, the relevance of instrument standardization and consistency stands out for scientific and clinical advancement in different areas and age groups,^32-35^ while recent studies reinforce the need for rigorous validation processes in different contexts to ensure clinical applicability supported by scientific evidence.^36^

### Stratified form (each instrument item)

The inclusion of κw and Gwet’s AC2 coefficient as sensitivity analyses, in response to peer review, reinforced the conclusions drawn from Kendall’s τ and gross agreement. In items where conventional chance-corrected coefficients (κ, κw) were undermined by the prevalence paradox^37^—that is, items with one category dominating the marginal distribution—Gwet’s AC2 consistently yielded estimates above 0.80, indicating substantial agreement that might otherwise be obscured. This convergence between κw and AC2 in items with balanced variability, combined with the divergence in items with low variability, illustrates a well-documented phenomenon in the literature on ordinal agreement^22,23,37^and supports the interpretation that the ICR/DMO presents satisfactory reliability across the range of items, including those with prevalence-dominant categories. Bowker’s test of symmetry further confirmed the absence of systematic directional bias between raters in the vast majority of testable items, indicating that the agreement reflected by Kendall’s τ is consistent with concordance rather than merely ordinal association.

The reliability results obtained from the stratified analysis showed that some items had no variance, such as “Family history (regarding the complaint)” and “Respiratory problems,” which prevented the calculation of Kendall’s τ, but revealed high gross agreement, indicating uniformity between judgments. This result may be explained by the low clinical prevalence of these conditions or by the tendency of responses to be concentrated in a single category (classification).

Other items, such as “Eating meals,” “Current diet – type and acceptance,” and “Hearing,” showed high percentages of agreement (≥83.3%), although without statistical significance, possibly due to low response variability and the consequent reduction in the inferential power of the coefficient. The item “Gestational and perinatal complications” showed lower agreement (66.7%), which may reflect the heterogeneity of information reported by caregivers, making the classification more susceptible to divergences between raters.

Such results are in line with phenomena already described in the literature, such as the paradox between high gross agreement and low statistical coefficients, in which the asymmetrical distribution of categories or the excess of ties can artificially reduce association rates.^37^ These findings suggest the need for broader and more diverse samples in future studies, as well as additional steps such as discriminant validity assessment.^33^

Regarding the ICR/DMO application/recording time, the instrument showed relevant advantages by demonstrating significantly short average application/recording times, which favors its applicability in different speech-language pathology contexts, whether in private or public services. The standard deviation (SD) values were expected, since the number of applicable items increases in older age groups, and these children commonly present more complaints or apparent changes,^21,38^ requiring longer reporting time from caregivers during the application of the instrument. Nevertheless, at all ages, the estimated time appears highly relevant for a OMD screening instrument.

Therefore, the ICR/DMO was validated as a screening tool to be applied by speech-language pathologists to caregivers of infants and/or preschoolers.

## Study limitations and strengths

Among the study limitations, despite the availability of the main researcher, the difficulty some caregivers experienced in attending daycare centers on the scheduled days and times for research participation stood out. Another limitation refers to some instrument items showing low response variability, making it impossible to calculate certain inferential coefficients. In these cases, the analysis was complemented with the gross agreement percentage, which is a methodologically valid approach.

Another methodological limitation refers to the inter-rater design: each secondary rater (A2a, A2b, and A2c) evaluated a single age group, meaning that no rater contributed observations across all three strata. Consequently, the inter-rater estimates within each group are pairwise (between the primary rater A1 and the corresponding A2) and reflect agreement between two evaluators within a specific age range, rather than agreement across a panel of raters evaluating the same cases. This design was adopted to balance the workload among raters with stratum-specific clinical expertise, but it does not enable the separation of potential rater-specific effects from age-group effects in the reliability estimates. Future studies should consider designs in which all raters evaluate a common subset of cases across age groups, enabling the estimation of multi-rater reliability coefficients such as Fleiss’ kappa or Krippendorff’s alpha.

A further methodological consideration concerns the source of the secondary assessments: raters A2a, A2b, and A2c, as well as the A1 retest, were all based on the same audio recordings of each interview. While this approach maximizes standardization and isolates rater scoring behavior from contextual variability (e.g., environment, caregiver mood, examiner cues), it inherently reduces the variability that would be present in independent face-to-face administrations. Therefore, the reported reliability estimates should be interpreted as evidence of scoring reliability under standardized listening conditions, rather than as overall inter-administration reliability under naturalistic clinical conditions. Future studies should incorporate independent live administrations of the ICR/DMO by different raters to estimate reliability under field conditions.

Among the contributions of this study, progress in the field of OM stands out, strengthening scientific evidence related to the first risk screening instrument for OMD aimed at infants and preschoolers. By specifically investigating the reliability of the ICR/DMO, this study expands the body of evidence regarding its psychometric properties, complementing previous validation steps and consolidating its consistency for clinical and scientific use.^7,8,11,15,26^

The findings reinforce the stability and precision of the measurements obtained with the ICR/DMO, which are essential aspects for its application in different speech-language pathology contexts. The score-based structure favors standardized risk classification and supports professional decision-making while maintaining consistency with established theoretical frameworks. This potential makes it possible to define epidemiological profiles by offering technical support for strategies in public health policies aimed at the early identification of orofacial myofunctional risks and their progression.

Additionally, the ICR/DMO aligns with international commitments aimed at promoting health and child development by contributing to the early identification of risk factors and strengthening evidence-based practices. Its use also offers educational potential, supporting teaching and research activities in speech-language pathology.^39^

The application of the instrument remains aligned with the person- and family-centered care model, as it incorporates information provided by the caregivers into the evaluation process. This approach favors active family participation, expands the understanding of the child’s context, and enhances the identification of orofacial myofunctional risks by integrating technical-scientific knowledge with everyday experience.^40^

Finally, we recognize the need for future studies to advance the definition of the remaining ICR/DMO validation steps.

## Conclusion

The reliability stage of the psychometric validation process of the ICR/DMO, intended for risk classification for DMO in infants and preschoolers, was verified.

The reliability analyses of the ICR/DMO—based on Kendall’s τ, weighted Kappa, Gwet’s AC2, and ICC(2,1)—provided convergent evidence of inter-rater consistency and intra-rater stability across all three age groups, ranging from moderate to excellent agreement. For items with limited marginal variability, Gwet’s AC2 supported the interpretation of substantial observed agreement. These findings support the use of the ICR/DMO as a screening tool for orofacial myofunctional risk in infants and preschoolers, while acknowledging the heterogeneity of item-level results and the need for further validation studies in samples with greater clinical variability.

## References

[1] Assis HS, Alves MV, Barreto ID, Rezende GE, Medeiros AM (2023). Perfil os fonoaudiólogos com formação em motricidade orofacial no Brasil. Audiol. Commun Res.

[2] Martins FS, Silva MF, Souza DS, Farias RR, Ramos PF (2021). Malocclusion and speech therapy and associated factors: integrative review. Res Soc Dev.

[3] Rezende GE, Santos JA, Oliveira EB, Barreto ID, Guedes-Granzotti RB, Medeiros AM (2025). Speech therapy instruments for tracking and screening: scope review. Distúrb Comun.

[4] Melo AT, Barbosa GD, Jesus EM, Matos AL, Santos EM, Barreto ÍD (2022). Clinical history speech-language pathology protocols: integrative review. Audiol Commun Res.

[5] Medeiros AM, Marchesan IQ, Genaro KF, Barreto ÍD, Berretin-Felix G (2022). MMBGR protocol - infants and preschoolers: instructive and orofacial myofunctional clinical history. CoDAS.

[6] Santos AS, Goes YD, Assis HS, Alves MV, Melo AT, Barbosa GD (2024). alidity based on the response processes of the MMBGR Protocol Infants and preschoolers: instructional and orofacial myofuncional clinical history. CoDAS.

[7] Fujinaga CI, Zamberlan NE, Rodarte MD, Scochi CG (2007). Reliability of an instrument to assess the readiness of preterm infants for oral feeding. Pró-Fono R Atual Cient.

[8] Martinelli RL, Marchesan IQ, Lauris JR, Honório HM, Gusmão RJ, Berretin-Felix G (2016). Validity and reliability of the neonatal tongue screening test. Rev CEFAC.

[9] Medeiros AM, Nascimento HS, Santos MK, Barreto ID, esus EM (2018). Content analysis and appearance of the speech therapy protocol of accompanying - breastfeeding. Audiol Commun Res.

[10] Medeiros AM, Nobre GR, Barreto ID, Jesus EM, Folha GA, Matos AL (2021). Expanded Protocol of Orofacial Myofunctional Evaluation with Scores for Nursing Infants (6-24 months) (OMES-E Infants). CoDAS.

[11] Medeiros AM, Marchesan IQ, Genaro KF, Barreto ÍD, Berretin-Felix G (2022). MMBGR Protocol - Infants and Preschoolers: Myofunctional Orofacial Clinic Examination. CoDAS.

[12] Felício CM, Ferreira CL (2008). Protocol of orofacial myofunctional evaluation with scores. Int J Pediatr Otorhinolaryngol.

[13] Genaro KF, Berretin-Felix G, Rehder MI, Marchesan IQ (2009). Orofacial myofunctional evaluation: MBGR protocol. Rev CEFAC.

[14] Lima MM, Cordeiro AA, Queiroga BA (2021). Developmental stuttering screening instrument: development and content validation. Rev CEFAC.

[15] Pernambuco L, Espelt A, Magalhães HV, Lima KC (2017). Recommendations for elaboration, transcultural adaptation and validation process of tests in Speech, Hearing and Language Pathology. CoDAS.

[16] Bonett DG, Wright TA (2000). Sample size requirements for estimating Pearson, Spearman and Kendall correlations. Psychometrika.

[17] Koo TK, Li MY (2016). A guideline of selecting and reporting intraclass correlation coefficients for reliability research. J Chiropr Med.

[18] Teker M, Yildiz B, Teker K (2015). Examining cronbach alpha, theta, omega reliability coefficients according to sample size. J Mod Educ Rev.

[19] Shou Y, Olney J (2020). Assessing a domain-specific risk-taking construct: a meta-analysis of reliability of the DOSPERT scale. Judgm Decis Mak.

[20] Zaki R, Bulgiba A, Nordin N, Azina Ismail N (2013). A Systematic review of statistical methods used to test for reliability of medical instruments measuring continuous variables. Iran J Basic Med Sci.

[21] Medeiros AM, Assis HS, Alves MV, Santana YF, Silva-Filho WJ, Barreto ID (2023). Orofacial myofunctional aspects of nursing infants and preschoolers. Int Arch Otorhinolar.

[22] De Raad A, Warrens MJ, Bosker RJ (2021). A comparison of reliability coefficients for ordinal rating scales. J Classif.

[23] Vanbelle S, Hernandez Engelhart C, Blix E (2024). A comprehensive guide to study the agreement and reliability of multi-observer ordinal data. BMC Med Res Methodol.

[24] R Core Team (2023). R: a language and environment for statistical computing.

[25] The jamovi project (2022). jamovi.

[26] American Educational Research Association, American Psychological Association, National Council on Measurement in Education (2014). Standards for educational and psychological testing.

[27] Rocha BR, Behlau M, Madazio G, Moreti F, Azevedo R, Brasil OO (2024). Validation and cutoff value of the Brazilian version of the Evaluation of the Ability to Sing Easily (EASE). J Voice.

[28] Mokkink LB, Vet HC, Prinsen CA, Patrick DL, Alonso J, Bouter LM (2018). COSMIN risk of bias checklist for systematic reviews of patient-reported outcome measures. Qual Life Res.

[29] Elsman EB, Mokkink LB, Terwee CB, Gagnier JJ, Tricco AC, Baba A (2024). Guideline for reporting systematic reviews of outcome measurement instruments (OMIs): PRISMA-COSMIN for OMIs 2024. J Clin Epidemiol.

[30] Aguiar EL, Barbosa NS, Neves TM (2023). Atypical swallowing as a form of postnatal development of oral function: literature review. Res Soc Dev.

[31] Scataglini S, Abts E, Bocxlaer CV, Bussche MV, Meletani S, Truijen S (2024). Accuracy, validity, and reliability of markerless camera-based 3D motion capture systems versus marker-based 3D motion capture systems in gait analysis: a systematic review and meta-analysis. Sensors (Basel).

[32] Etges CL, Barbosa LR, Cardoso MC (2020). Development of the Pediatric Dysphagia Risk Screening Instrument (PDRSI). CoDAS.

[33] Botura C, Alves GS, Bernardi AC, Ribas LP (2024). Phonological assessment instrument: evidence of construct validity. CoDAS.

[34] Rocha MC, Nogueira BF, Nunes FB, Medeiros AM (2024). Self-perception of voice, hearing, and general health in screening for voice changes in older women. CoDAS.

[35] Queiroga CA, Queiroga BA, Almeida DP, Cordeiro AA (2024). Development and content validation of the Communication Screening Instrument - IRC-36. Rev CEFAC.

[36] Cruchinho P, López-Franco MD, Capelas ML, Almeida S, Bennett PM, Silva MM (2024). Translation, cross-cultural adaptation, and validation of measurement instruments: a practical guideline for novice researchers. J Multidiscip Healthc.

[37] Minozzi S, Cinquini M, Gianola S, Gonzalez-Lorenzo M, Banzi R (2022). Kappa and AC1/2 statistics: beyond the paradox. J Clin Epidemiol.

[38] Chang MC, Liu HY, Huang ST, Chen HL (2022). Study of orofacial function in preschool children born prematurely. Children (Basel).

[39] Organização das Nações Unidas (2015). Transformando nosso mundo: a Agenda 2030 para o desenvolvimento sustentável.

[40] Melis MT, Apolônio AL, Santos LC, Ferrari DV, Abramides DV (2022). Social skills training in Speech-Language Pathology and Audiology: students' perception. Rev CEFAC.

